# Fishes can use axial muscles as anchors or motors for powerful suction feeding

**DOI:** 10.1242/jeb.225649

**Published:** 2020-09-18

**Authors:** Ariel L. Camp, Aaron M. Olsen, L. Patricia Hernandez, Elizabeth L. Brainerd

**Affiliations:** 1 Department of Musculoskeletal and Ageing Science, Institute of Life Course and Medical Sciences, University of Liverpool, Liverpool L7 8TX, UK; 2 Department of Ecology and Evolutionary Biology, Brown University, Providence, RI 02912, USA; 3 Department of Biological Sciences, The George Washington University, Washington, DC 20052, USA

**Keywords:** Hypaxial, Epaxial, Craniovertebral, Pectoral girdle, XROMM

## Abstract

Some fishes rely on large regions of the dorsal (epaxial) and ventral (hypaxial) body muscles to power suction feeding. Epaxial and hypaxial muscles are known to act as motors, powering rapid mouth expansion by shortening to elevate the neurocranium and retract the pectoral girdle, respectively. However, some species, like catfishes, use little cranial elevation. Are these fishes instead using the epaxial muscles to forcefully anchor the head, and if so, are they limited to lower-power strikes? We used X-ray imaging to measure epaxial and hypaxial length dynamics (fluoromicrometry) and associated skeletal motions (XROMM) during 24 suction feeding strikes from three channel catfish (*Ictalurus punctatus*). We also estimated the power required for suction feeding from oral pressure and dynamic endocast volume measurements. Cranial elevation relative to the body was small (<5 deg) and the epaxial muscles did not shorten during peak expansion power. In contrast, the hypaxial muscles consistently shortened by 4–8% to rotate the pectoral girdle 6–11 deg relative to the body. Despite only the hypaxial muscles generating power, catfish strikes were similar in power to those of other species, such as largemouth bass (*Micropterus salmoides*), that use epaxial and hypaxial muscles to power mouth expansion. These results show that the epaxial muscles are not used as motors in catfish, but suggest they position and stabilize the cranium while the hypaxial muscles power mouth expansion ventrally. Thus, axial muscles can serve fundamentally different mechanical roles in generating and controlling cranial motion during suction feeding in fishes.

## Abstract (precis)

**Highlighted Article:** Channel catfish use their dorsal body muscles to stabilize the head during suction feeding, while the ventral body muscles power mouth expansion.

## INTRODUCTION

**Fig. 1.**
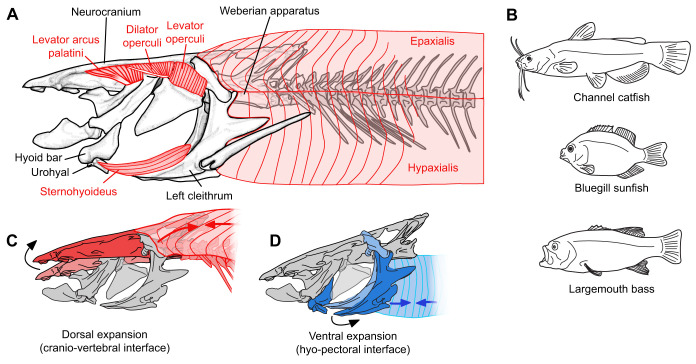
**Body shape and anatomy of the feeding apparatus of channel catfish *Ictalurus punctatus*.** (A) Lateral view of the neurocranium and Weberian apparatus, pectoral girdle, left-side bones and muscles of the cranial expansion system, and the cranial half of the axial muscles and vertebral column. Note that the white portions of the levator arcus palatini and dilator operculi show the tendinous portions of these muscles. (B) Whole-body shape of the channel catfish compared with previously studied species: bluegill sunfish (*Lepomis macrochirus*) and largemouth bass (*Micropterus salmoides*). (C) Dorsal and (D) ventral expansion modules, which could be used to increase the volume of the mouth during suction feeding. These also illustrate the dorsal, cranio-vertebral interface between the head and the body, and the ventral, hyo-pectoral interface.

In ray-finned fishes the postcranial bones and muscles are an important part of the feeding apparatus. In most species the bones and muscles of the vertebral column and pectoral girdle attach directly to the head, forming dorsal (cranio-vertebral) and ventral (hyo-pectoral) interfaces between the head and body (Camp, 2019; Day et al., 2015; Liem, 1980; Muller, 1987; Tchernavin, 1948). These anatomical connections allow the body muscles to contribute to cranial motions, such as expanding the mouth cavity during suction feeding (Fig. 1). Suction feeding is the most common feeding mode in fishes, and relies on rapid mouth expansion to increase volume and decrease pressure inside the mouth cavity, generating high-velocity fluid flows that accelerate entrained food items into the mouth (Ferry-Graham and Lauder, 2001; Jacobs and Holzman, 2018). Both postcranial interfaces can act on the cranial linkage system of the skull to increase the volume of the mouth cavity (Aerts et al., 1987; Alexander, 1970; Camp et al., 2015; Carroll and Wainwright, 2006; Muller, 1987). Dorsally, the epaxial muscles can act through the cranio-vertebral interface to dorsally rotate (elevate) the neurocranium relative to the vertebral column (Fig. 1C). These are the only muscles that cross the craniovertebral joint and can generate cranial elevation. Ventrally, the hypaxial muscles can act through the hyo-pectoral interface to retract the pectoral girdle (Fig. 1D), which in turn depresses and retracts the hyoid apparatus and lower jaw (Van Wassenbergh et al., 2005b). Ventral expansion can also be generated by the sternohyoideus muscle (Fig. 1A) shortening to retract the hyoid apparatus relative to the pectoral girdle. The mouth cavity can also expand laterally through the abduction of bony elements to increase the width of the mouth cavity. Lateral expansion can be generated by direct muscle action, as well as motion transmitted through bony linkages from the dorsal and ventral expansion modules (Liem, 1980; Muller, 1989). The dorsal and ventral expansion modules may function independently, or their motions may be coupled as they are connected to each other through the cranial linkage system (Muller, 1987).

The hypaxial and epaxial muscles can contribute substantially to expanding the mouth cavity (buccal and opercular cavities) during suction feeding. The power for this mouth expansion is generated by muscles actively producing force and positive (shortening) velocity, as power is the product of force and velocity. The axial muscles have the appropriate anatomy to generate mouth expansion motions, as described above, and are active during suction feeding in many species (Grubich, 2001). Recent studies of largemouth bass, *Micropterus salmoides* (Camp and Brainerd, 2014), and bluegill sunfish, *Lepomis macrochirus* (Camp et al., 2018), have shown that large regions of hypaxial and epaxial muscles shorten during suction feeding, and therefore generate power for mouth expansion. Moreover, these axial muscles generated at least 90% of the power required for high-performance suction feeding strikes in these species (Camp et al., 2015, 2018). It is reasonable to expect that fishes with similar feeding behaviors and kinematics – including cranial elevation and pectoral girdle retraction – also use both epaxial and hypaxial muscles as motors to power suction feeding. However, not all fishes both elevate the head and retract the pectoral girdle during suction feeding (Alexander, 1970; Van Wassenbergh et al., 2009).

Clariid catfishes are one example of suction feeding fishes where cranial elevation may be minimal or absent (Van Wassenbergh et al., 2009), and epaxial muscles are therefore unlikely to generate power for suction feeding. Previous studies have observed variable cranial elevation – from minor depression to 25 deg elevation – in clariid catfishes during suction feeding (Van Wassenbergh et al., 2004, 2009). The sternohyoideus lengthened during suction feeding in two species (and therefore could not contribute substantial power), and only shortened during the first half of mouth expansion in a third species (Van Wassenbergh et al., 2007b). But pectoral girdle retraction was consistent and substantial, leading to the conclusion that the hypaxial muscles provided most of the power for mouth expansion (Van Wassenbergh et al., 2005b; Van Wassenbergh et al., 2007b). This suggests that, unlike bass and sunfish, clariid catfishes are using primarily the hypaxial muscles, and not the epaxial muscles, to power mouth expansion.

Instead, the epaxial muscles and cranio-vertebral interface in clariid catfishes may act as an anchor to control the position of the head and stabilize the dorsal ‘roof’ of the mouth. Anchoring the neurocranium during suction feeding could provide a stable attachment site for cranial muscles and also resist the sub-ambient pressures in the expanding mouth cavity (Camp, 2019). Otherwise, if the neurocranium were free to move, the sub-ambient pressure resulting from ventral expansion would pull the neurocranium towards the center of the mouth cavity, resulting in the whole head rotating ventrally rather than expanding (Carroll et al., 2004; Muller, 1987). In functioning as an anchor to maintain the position of the head, the cranio-vertebral interface may still undergo motion (e.g. to move the mouth into an optimal position over the food) even though the expaxial muscles are not powering mouth expansion. Indeed, these ‘anchor’ and ‘motor’ functions represent extrema of a functional continuum proposed for the postcranial interfaces during feeding (Camp, 2019). In reality, the cranio-vertebral interface may combine anchor and motor functions throughout feeding, and measurements of joint and muscle motion are needed to determine its role. However, epaxial muscle length and neurocranium motion have not been measured in clariid catfish to test this. Anchor functions of the cranio-vertebral interface have been difficult to confirm in fishes because it requires measuring dorsal and ventral expansion independently of each other (i.e. relative to the body), and measuring length changes across the large axial muscles.

If some fishes are using the epaxial muscles and cranio-vertebral interface as anchors during suction feeding, can they still generate powerful mouth expansion? Mouth expansion has been estimated to require substantial power – to a maximum of 300–400 W kg^−1^ in bluegill sunfish (Camp et al., 2018) – and all else being equal, greater muscle masses can generate more power. Therefore, the massive, fast-fibered epaxial muscles have the potential to generate substantial power for suction feeding. But without cranial elevation and muscle shortening, the epaxial muscles cannot generate positive power. Are fish like the clariid catfishes then limited to less-powerful suction feeding strikes, compared with species like largemouth bass and bluegill sunfish that use both axial muscles to power suction feeding?

This study used channel catfish (*Ictalurus punctatus*, family Ictaluridae) to test the hypotheses that (1) the epaxial muscles and cranio-vertebral interface position and stabilize the cranial linkage dorsally during suction feeding, while the hypaxial muscles power mouth expansion ventrally and that (2) as a result, catfish are limited to less-powerful suction feeding strikes than largemouth bass and bluegill sunfish. Like clariid catfishes, *I. punctatus* has a dorsoventrally compressed head and uses ventral expansion, including hypaxial muscle-powered pectoral girdle retraction, during suction feeding (Olsen et al., 2019). We used X-ray reconstruction of moving morphology (XROMM) (Brainerd et al., 2010) to measure cranial elevation and pectoral girdle retraction independently and relative to the body, and fluoromicrometry (Camp et al., 2016) to measure length changes throughout the epaxial and hypaxial muscles. The raw data for these XROMM animations and fluoromicrometry come from an existing dataset of X-ray videos, intra-oral pressure and digital bone models (Olsen et al., 2019). Additionally, we estimated mouth expansion power from measurements of the rate of mouth volume change from the XROMM animations (using a dynamic endocast) and intra-oral pressure measurements. With these data, we determined the role of the epaxial muscles and cranio-vertebral interface during suction feeding and how this impacts the fish's ability to generate powerful strikes. Examining the diversity of axial muscle functions during suction feeding will provide a better understanding of the evolution and functional morphology of this feeding behavior, and how it has been successfully employed across the spectacular morphological diversity of ray-finned fishes.

## MATERIALS AND METHODS

We used X-ray video and CT data from three channel catfish (*Ictalurus punctatus*, Rafinesque 1818): Cat1 (individual 1; standard length SL=32 cm), Cat2 (individual 2; SL=31 cm) and Cat5 (individual 3; SL=38 cm), collected as part of a previous study (Olsen et al., 2019). We summarize the methods briefly below, but a full description of surgical procedures and X-ray video collection is in Olsen et al. (2019). Catfish were obtained from Osage Catfisheries, Inc. (Osage Beach, MO, USA) and maintained at Brown University on a diet of sinking pellets and earthworms. All experimental procedures were approved by the Brown University Institutional Animal Care and Use Committee.

### Marker implantation

Each fish was anesthetized with buffered tricaine methanesulfonate and markers (0.5–0.8 mm diameter tantalum spheres) were surgically implanted into the fish's bones and muscles. The neurocranium, urohyal and the left lower jaw, suspensorium, operculum, ceratohyal, cleithrum and post-temporal bones were marked, along with the epaxial and hypaxial muscles. Muscle markers were implanted superficially and just to the left of the midsagittal plane in the epaxial muscles (4 markers) and hypaxial muscles (4–5 markers), and mid-belly in the sternohyoideus (2–3 markers). Three additional, deep epaxial markers were used with 3 superficial epaxial markers to define a body plane (Camp and Brainerd, 2014). A cannula to hold the pressure probe was implanted in the ethmo-frontal region of the neurocranium. All fish recovered fully within 3 days, resuming aggressive feeding with no signs of the implants causing distress or impeding normal behavior.

### Feeding trials

**Fig. 2.**
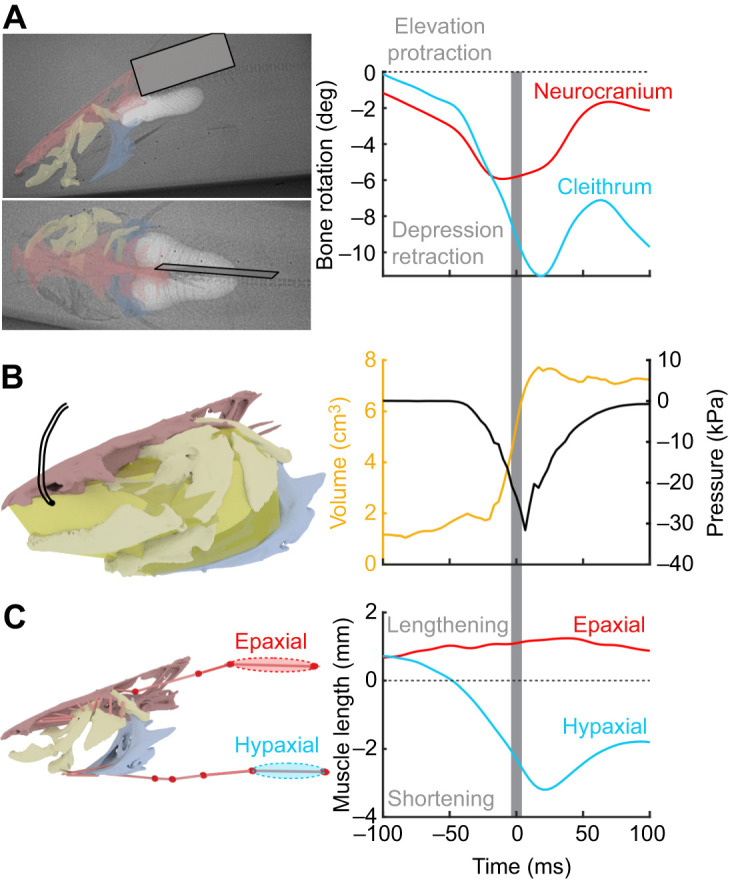
**Methods for measuring bone and muscle kinematics and estimating mouth expansion power during suction feeding.** All measurements are from a sample strike from individual Cat5 and are shown relative to their initial value prior to the strike, with time relative to the time of peak gape. The gray bar shows the period of peak (within 25% of maximum) mouth expansion power. (A) Biplanar X-ray videos (left lateral view and dorsal view) with XROMM animated bone models (neurocranium in red, cleithrum in blue, body plane in gray) superimposed. Dorsoventral rotation of the neurocranium (red) and cleithrum (blue) was measured relative to the body plane, with negative values indicating cranial depression and cleithrum retraction. (B) Mouth (buccal and opercular) volume was estimated from the animated skeleton using a dynamic endocast (yellow), while pressure was recorded with an intra-oral transducer (black). (C) Cranial muscle lengths (pale red cylinders) were measured from virtual landmarks on animated bones, while axial muscle lengths were measured from intramuscular markers with fluoromicrometry (red spheres and cylinders). Sample muscle lengths are shown from subregions of the epaxial muscles (red line and highlighted region) and hypaxial muscles (blue line and highlighted region), with positive values indicating lengthening and negative values indicating shortening.

High-speed, biplanar X-ray videos were collected at 300 frames s^−1^ from each fish during suction feeding (Fig. 2A), as described in Olsen et al. (2019). Intra-oral pressure was measured with a SPR-407 Mikro-tip pressure probe (Millar Instruments, Houston, TX, USA) inserted into the neurocranial cannula (Fig. 2B), recording at 1000 Hz with PowerLab and LabChart 7.2.2 (ADInstruments, Colorado Springs, CO, USA). The pressure probe was calibrated daily, and synchronization tests confirmed no offset between pressure and video recording. Each fish was recorded capturing half or whole live worms (>25 mm), dead squid pieces (up to 25 mm) or sinking pellets (approximately 5 mm), and the trials with the greatest sub-ambient pressure (almost exclusively worm trials) from each individual were selected for further analysis. Video, pressure and calibration data are stored with their essential metadata on the XMAPortal (http://xmaportal.org) in the study ‘Catfish suction feeding’, with the permanent identifier BROWN61, in accordance with best practices for video data management in organismal biology (Brainerd et al., 2017).

### Morphological data

Computed tomography (CT) scans of each fish were taken post-mortem on a FIDEX CT Scanner (Animage, Pleasanton, CA, USA), with 480×480 pixel resolution and 0.173 mm slice thickness. Right-side muscles (levator arcus palatini, levator operculi, dilator operculi, sternohyoideus, epaxial muscles, hypaxial muscles; see Fig. 1) were dissected out and weighed. Epaxial and hypaxial muscle mass included only the marked region of each muscle. On the left side of the fish, the bony attachment sites of the cranial muscles were exposed and the origin and insertion of three fibers were marked before CT scanning (Camp et al., 2018). Polygonal mesh models of each marked bone and all bone and muscle markers were generated in Horos (v3.3.5; Horos Project; horosproject.org) and edited in Geomagic 2013 (Research Triangle Park, NC, USA).

### Pressure data

Intra-oral pressure recordings were exported from LabChart, and all further analysis done with custom-written scripts in MATLAB (R2019a; The Mathworks, Natick, MA, USA) following the methods of Camp et al. (2018). Pressure data were filtered (Butterworth, low-pass, 300 Hz cutoff), converted from mV to Pa, and re-calculated relative to the initial, ambient pressure prior to the start of the strike. Therefore, negative pressure values indicate sub-ambient intra-oral pressure (Fig. 2B).

### XROMM animation

Undistortion transforms were generated, the X-ray videos were calibrated, and markers tracked to calculate the 3D coordinates of each bone and muscle marker using XMALab 1.5-1.5.2 (Knörlein et al., 2016). Software and instructions are available at https://bitbucket.org/xromm/xmalab. Marker tracking precision, measured as the mean of the standard deviation of intraosseus markers, averaged 0.08 mm across all bones. Bone marker coordinates were combined with marker coordinates from the CT scan to calculate and filter (Butterworth, low-pass, 40 Hz) the rigid body transformations of each marked bone in XMALab. For the body plane (Fig. 2A), rigid body transformations were calculated as though it was a single, rigid object based on the 6 epaxial markers (Camp and Brainerd, 2014). For the post-temporal and suspensorium, the implanted markers were restricted to a small portion of the bone and additional virtual landmarks were placed at joints between these bones and the neurocranium and animated with the neurocranium to more accurately calculate their rigid body transformations. Where necessary, marker-based animation was supplemented with scientific rotoscoping: manually aligning the bone model to match both X-ray images (Gatesy et al., 2010). These methods were used together to create a single skeletal animation for each strike in Maya 2018 (Autodesk, San Rafael, CA, USA) using custom-written MEL scripts (available at https://bitbucket.org/xromm/xromm_mayatools).

### Skeletal kinematics

**Fig. 3.**
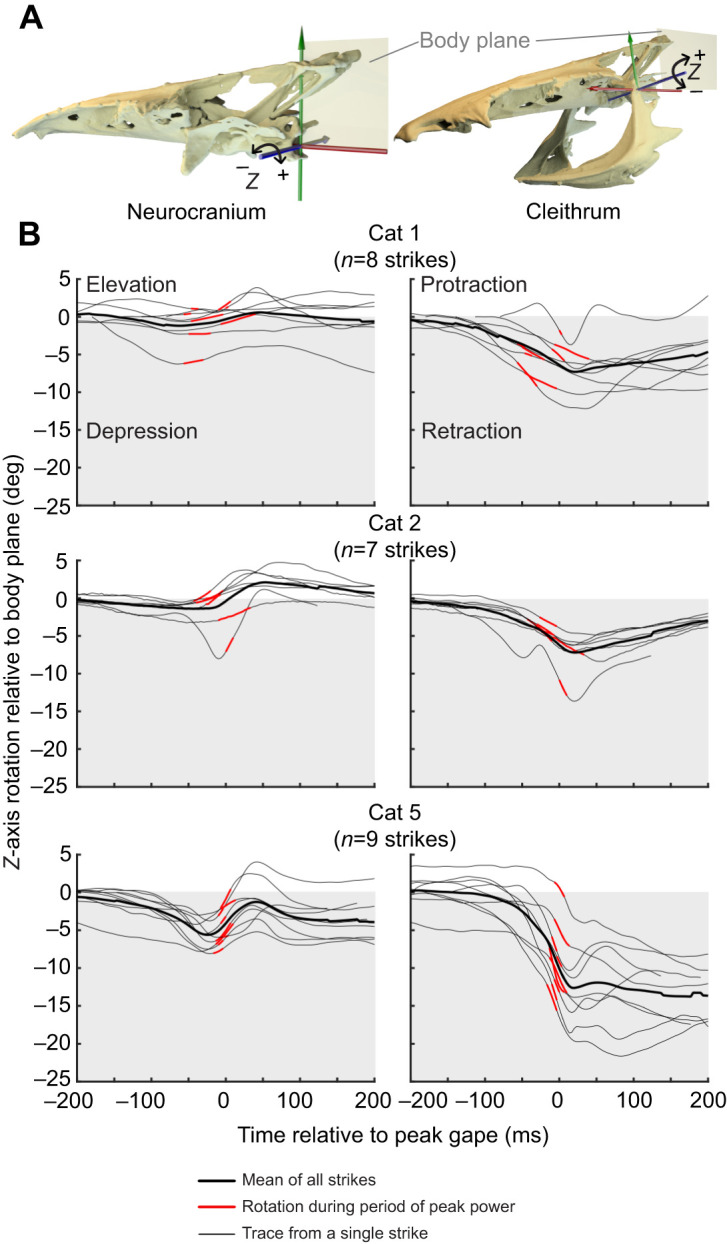
**Neurocranium and cleithrum rotations** **relative to the body plane.** (A) The primary rotations of each bone were measured about the *z*-axis (in blue) of their respective joint coordinate systems, using a *zyx* rotation order and following the right-hand rule. Bone models and the body plane (semi-transparent) are shown. (B) For each individual, *z*-axis rotations (relative to initial values) are shown for each strike (thin black lines), with the period during peak (within 25% of maximum) expansion power highlighted in red. Positive values (white plot area) indicate elevation or protraction and negative values (gray plot area) indicate depression or retraction. The mean rotation at each time point (for all strikes from that individual) is also shown (thick black line).

Neurocranium and cleithrum motion were measured relative to the body plane using joint coordinate systems (JCSs), as in Camp and Brainerd (2014) and Camp et al. (2018). For the neurocranium, an anatomical coordinate system (ACS) was created at the craniovertebral joint with the *x*-axis midsagittal and running rostro-caudally, the *y*-axis running dorsoventrally and the *z*-axis orthogonal to both. The ACS was duplicated, with one ACS attached to the neurocranium and one to the body plane, to create a JCS measuring neurocranium motion relative to the body plane (Fig. 3). For the cleithrum, the ACSs were aligned the same way and placed at the cleithrum–post-temporal joint to measure cleithrum motion relative to the body plane. Joint motions were measured as translations and rotations along and about each axis, following a *zyx* rotation order. For both JCSs, *z*-axis motion corresponds to translation toward the right side (positive) or left side (negative) and rotation dorsally (positive) or ventrally (negative). Thus, cranial elevation is measured as positive *z*-axis rotation and pectoral girdle retraction as negative *z*-axis rotation, relative to the body plane (Fig. 3). Gape was measured as the distance between the upper and lower jaws, using virtual landmarks attached to the rostral tip of the lower jaw and to the rostral tip of the neurocranium, in the midsagittal plane. To compare timings across strikes and individuals, we measured all times relative to the time of peak gape.

### Muscle length measurements

For the epaxial and hypaxial muscles, length changes were measured with fluoromicrometry (Fig. 2C; Movie 1): using the biplanar X-ray videos to measure the change in distance between radio-opaque, intramuscular markers (Camp et al., 2016). The *x*, *y* and *z* coordinates of the intramuscular markers were filtered with a Butterworth, low-pass filter at a 40 Hz cutoff. To measure length changes of muscles in the head, virtual landmarks were added to the animated bones in Maya at the attachment sites of three fibers within each muscle: levator operculi, sternohyoideus, levator arcus palatini and dilator operculi as in Camp et al. (2018). Whole-muscle length was calculated as the average length of all three fibers for each muscle at every frame (Movie 1). For parallel-fibered muscles with minimal tendons (sternohyoideus and levator operculi), these measurements represent both whole-muscle and muscle fiber length (Fig. 1). For muscles with substantial tendinous elements (levator arcus palatini, dilator operculi), these measurements represent the length of the muscle–tendon unit, not fiber length. Sternohyoideus length was also calculated from fluoromicrometry and compared with the lengths calculated from the XROMM animations to confirm that the two methods gave similar results. Calculation and analysis of muscle length changes were done in MATLAB with custom-written scripts (Camp and Brainerd, 2014; Camp et al., 2018).

For all muscles, strain was calculated as the percent change in length relative to the mean initial length (*L*_i_) across all strikes from each individual, with positive values indicating shortening. The strain rate, or shortening velocity, was calculated in initial lengths per second. For the axial muscles, strain was calculated within each subregion (i.e. between each pair of intramuscular markers) and across the whole region (i.e. between the cranial-most and caudal-most intramuscular markers). Maximum strain and strain rate during peak power (i.e. when expansion power was within 25% of its maximum value) were calculated for each muscle.

### Mouth volume measurements

Mouth volume was estimated dynamically throughout the suction strike by fitting an endocast to the space enclosed by the cranial skeleton using custom-written MATLAB and Maya scripts (available at https://bitbucket.org/ArielCamp/dynamicendocast). As in previous studies, virtual landmarks were placed along the medial surface of all left-side animated bones, and up to the midsagittal plane of the neurocranium and pectoral girdle (Camp et al., 2015). Based on dissections, the esophagus (caudal edge of the mouth cavity) was assumed to be in the frontal plane between the left and right cleithra. These landmarks were attached to the animated bones models, so they moved with the bones during the strike (Movie 2). The volume enclosed by the 3D landmarks was calculated in MATLAB (alphaShape function) using the 3D coordinates of all landmarks at each frame and an alpha value of 2. An obj file of the alpha shape for each frame was then imported into the XROMM animation to visually confirm the fit within the mouth cavity (Fig. 2B; Movie 2). The volume of this unilateral, dynamic endocast was doubled to estimate the total mouth cavity volume at each frame, assuming bilateral symmetry.

### Power estimates

The power required for mouth expansion was estimated from measurements of intra-oral pressure and rate of volume change using custom-written MATLAB scripts, as in Camp et al. (2018). Instantaneous expansion power at each frame was estimated as the product of the instantaneous pressure (Pa) and rate of volume change (m^3^ s^−1^) at that frame. Sub-ambient pressures were multiplied by −1 to give positive power values, and maximum expansion power was calculated for each strike. Instantaneous and maximum expansion power were normalized by the total mass of the cranial muscles (including the sternohyoideus) and the mass of the hypaxial muscles (marked region only) to determine the mass-specific outputs they would have to generate if they were the sole source of power for mouth expansion.

Our power values are estimates, as both the pressure and volume values have multiple sources of error (Camp et al., 2018). The dynamic endocast overestimates the absolute mouth volume because it does not take into account soft tissues or internal structures, although this overestimate should be constant throughout the strike and therefore have less impact on the rate of volume change. The single intra-oral pressure measurement does not reflect the variation in pressure throughout the mouth cavity during suction feeding (Muller et al., 1982; Van Wassenbergh, 2015). Modeling of a morphologically similar species, clariid catfishes, suggests the most sub-ambient pressures occur more caudally in the mouth cavity (Van Wassenbergh et al., 2005a, 2006a), and our pressure values likely underestimate the total sub-ambient pressure and mouth expansion power. Lastly, our estimates do not include the force required to overcome inertia or drag (Van Wassenbergh et al., 2015). While modeling studies of clariid catfishes suggest these would be small compared with those required to overcome sub-ambient pressure forces (Van Wassenbergh et al., 2005a), our values are likely underestimating expansion power.

## RESULTS

All catfish in this study used suction to capture prey items, with substantial variation among strikes and individuals. Therefore, we report mean and standard error values for each individual (*N*=8 strikes for Cat1, *N*=7 strikes for Cat2, and *N*=9 strikes for Cat5) below and in Tables 1 and 2. As previously described (Olsen et al., 2019), catfish were fed a variety of food items (pellets, squid pieces, live worms), but strikes on worms elicited the greatest sub-ambient pressures and suction power. We note that none of these foods were highly elusive and capable of escape responses, which can elicit greater and faster mouth expansion (Van Wassenbergh and De Rechter, 2011). As our goal was to analyze the most powerful suction feeding, we primarily used worm strikes (2 of 8 Cat1 strikes, 6 of 7 Cat2 strikes, and 9 of 9 Cat5 strikes were on worms). However, this unbalanced design did not allow us to test the impact of prey type on suction feeding, so we pooled data from all prey types for each individual. Pectoral girdle retraction and hypaxial shortening (over the cranial-most hypaxial subregion) were reported previously, although calculated from the X-ray videos using slightly different methods (Olsen et al., 2019). We include them here for context and comparison with the new measurements of the neurocranium, epaxial muscles and more caudal subregions of the hypaxial muscles.

**Table 1.**
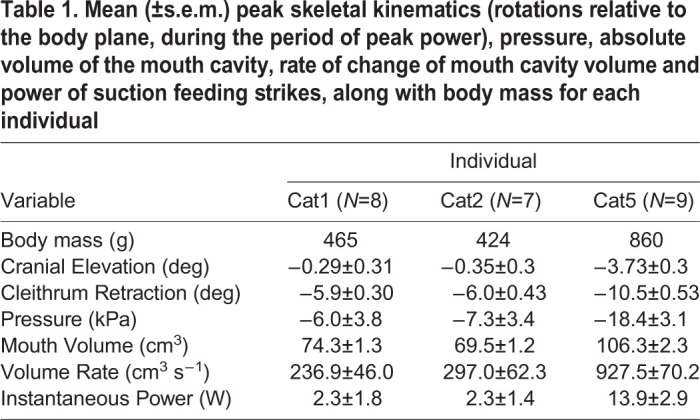
**Mean (±s.e.m.) peak skeletal kinematics (rotations relative to the body plane, during the period of peak power), pressure, absolute volume of the mouth cavity, rate of change of mouth cavity volume and power of suction feeding strikes, along with body mass for each individual**

**Table 2.**
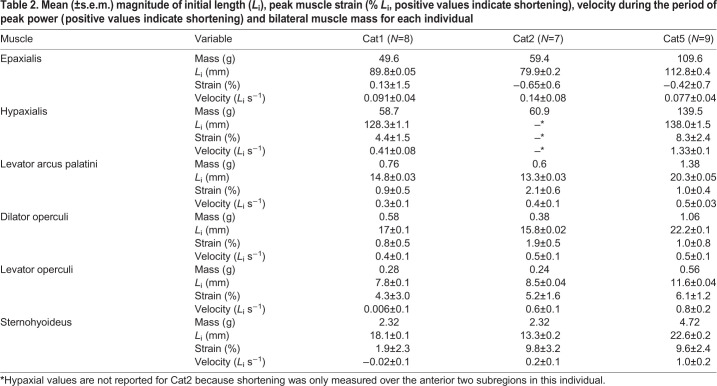
**Mean (±s.e.m.) magnitude of initial length (*L*_i_), peak muscle strain (% *L*_i_, positive values indicate shortening), velocity during the period of peak power (positive values indicate shortening) and bilateral muscle mass for each individual**

### Cleithrum and neurocranium kinematics

The cleithrum consistently retracted relative to the body plane, rotating caudoventrally and in the negative direction about the *z*-axis of the cleithrum JCS (Fig. 3, Table 1). Cleithrum retraction usually occurred during the period of peak power (Fig. 3), although it reached its peak just after this period. Dorsoventral rotation of the neurocranium relative to the body plane was small (usually <5 deg) and included both cranial elevation and depression (Fig. 3). In one individual, Cat5, the neuro cranium was consistently depressed (mean peak of −6.3±0.5 deg) and then elevated (mean peak of 1±0.5 deg). In this individual, cranial elevation did occur during peak power, although peak cranial elevation was reached later. For the remaining two individuals, dorsoventral cranial rotation was highly variable (Fig. 3). Where cranial elevation was present, its initial phase sometimes overlapped with the period of peak power. However, mean peak cranial elevation was low magnitude (Table 1) and occurred after the period of peak power.

### Muscle length changes

**Fig. 4.**
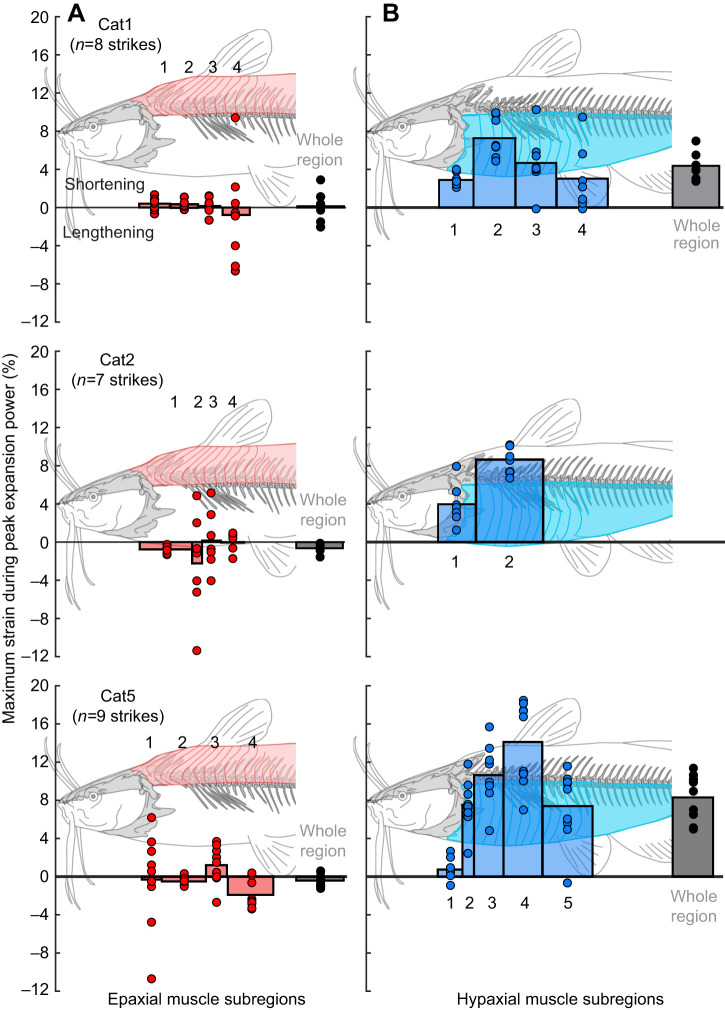
**Muscle strain within the epaxial and hypaxial muscles in each catfish.** Epaxial (A) and hypaxial (B) strain were calculated as percent change in length relative to the mean initial length of each subregion, with positive strain values representing muscle shortening. Maximum strain during peak expansion power is shown for each subregion, with both the per-strike strain (red or blue circles) and the mean strain across all strikes (red or blue bars). The schematic diagrams of the musculoskeletal system show the approximate location of each subregion, with the width of the bars approximating the cranio-caudal length of each subregion. Per-strike (black circles) and mean (gray bars) maximum strain values are also shown across the whole muscle region (from the cranial-most to caudal-most marker). Note that for Cat2, hypaxial markers were only visible in the two cranial-most subregions, so shortening was only measured for these subregions and no whole-region strain was calculated.

The epaxial muscles showed little or no shortening during mouth expansion. In all subregions measured, from the muscles' cranial attachments on the neurocranium to the caudal edge of the dorsal fin, mean strain during peak power production was near zero (Fig. 4A). Like neurocranium rotation, strain magnitude was highly variable across strikes and individuals, with both negative (muscle lengthening) and positive (muscle shortening) peaks (Fig. 4). Only subregion 3 in Cat5 showed small (up to 4% *L*_i_), but consistent positive strain during the period of peak power production (Fig. 4A).

In contrast, the hypaxial muscles consistently shortened over a region extending from their attachment on the cleithrum to at least the cranial edge of the anal fin (Fig. 4B). Across this entire region, the peak hypaxial strain during the period of peak power production averaged 4.4±1.5% and 8.3±2.4% for Cat1 and Cat5 (mean±s.e.m. % *L*_i_ for each individual, respectively). For Cat2, only the first two hypaxial subregions were visible and could be measured. Within the hypaxial regions investigated, the magnitude of strain varied across subregions. For all individuals, the first (cranial-most) subregion had the lowest magnitudes of peak strain (Fig. 4B). The largest strain magnitudes tended to occur in the second subregion in Cat1 and Cat2, but in the fourth subregion for Cat5. However, for all individuals and subregions, hypaxial shortening consistently occurred during the period of peak power production (Fig. 4B).

**Fig. 5.**
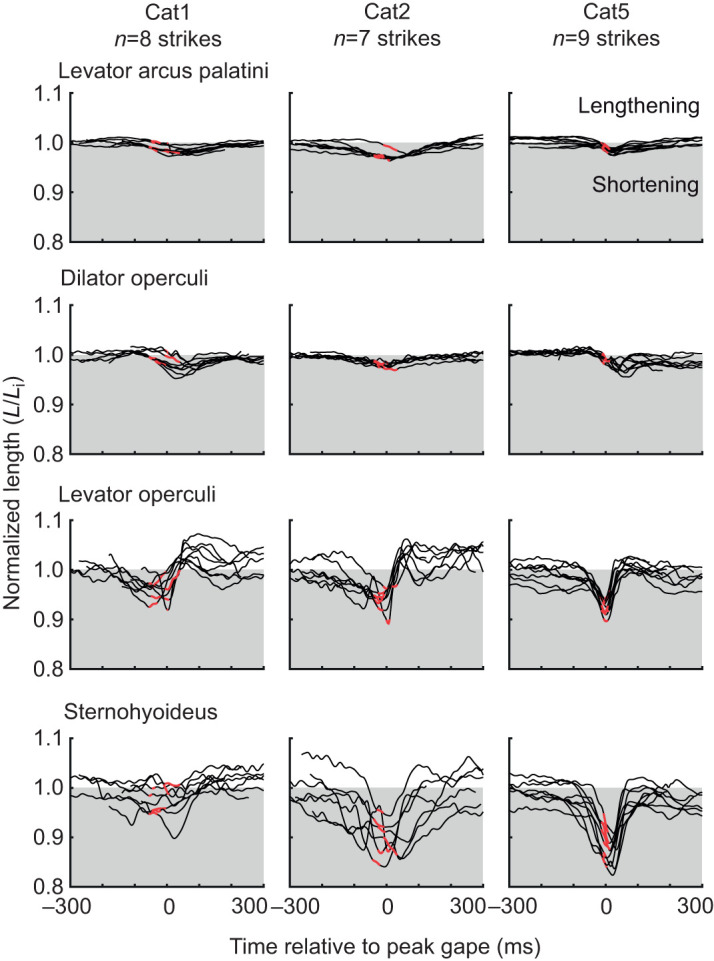
**Length change of the muscles of the head during mouth expansion in each catfish.** For each individual (columns) and each muscle (rows), muscle length was normalized by the mean initial length (*L*_i_) of each muscle and is shown as a function of time for all strikes from each individual (black lines). The strain occurring during peak (within 25% of maximum) expansion power is highlighted in red. Values below 1 indicate muscle shortening, and values above 1 indicate muscle lengthening, relative to *L*_i_. Note that for the levator arcus palatini and dilator operculi muscles, which had substantial tendinous portions (see Fig. 1), these lengths represent the whole muscle–tendon unit, while for the sternohyoid and levator operculi they correspond to fiber length.

Among the muscles of the head, only the sternohyoideus and levator operculi muscles showed substantial shortening during mouth expansion (Fig. 5). The sternohyoideus shortened consistently and substantially in Cat2 and Cat5, with mean maximum strains of nearly 10% *L*_i_ during the period of peak power (Table 2, Fig. 5). In Cat1, sternohyoideus length changes were much smaller – often less than 5% *L*_i_ – during the period of peak expansion power (Table 2, Fig. 5). The levator operculi shortened in all individuals during the period of peak expansion power, although again the magnitude was quite variable in Cat1 and Cat2 (Table 2, Fig. 5). For the levator arcus palatini and dilator operculi muscles, the whole muscle–tendon units did not change length substantially throughout the strike (Fig. 5). For both muscles the mean maximum strain during peak expansion power was <3% *L*_i_ (Table 2). However, we could not directly measure changes in the fiber length of these muscles as they had substantial tendons (Fig. 1A).

### Estimated mouth expansion power

**Fig. 6.**
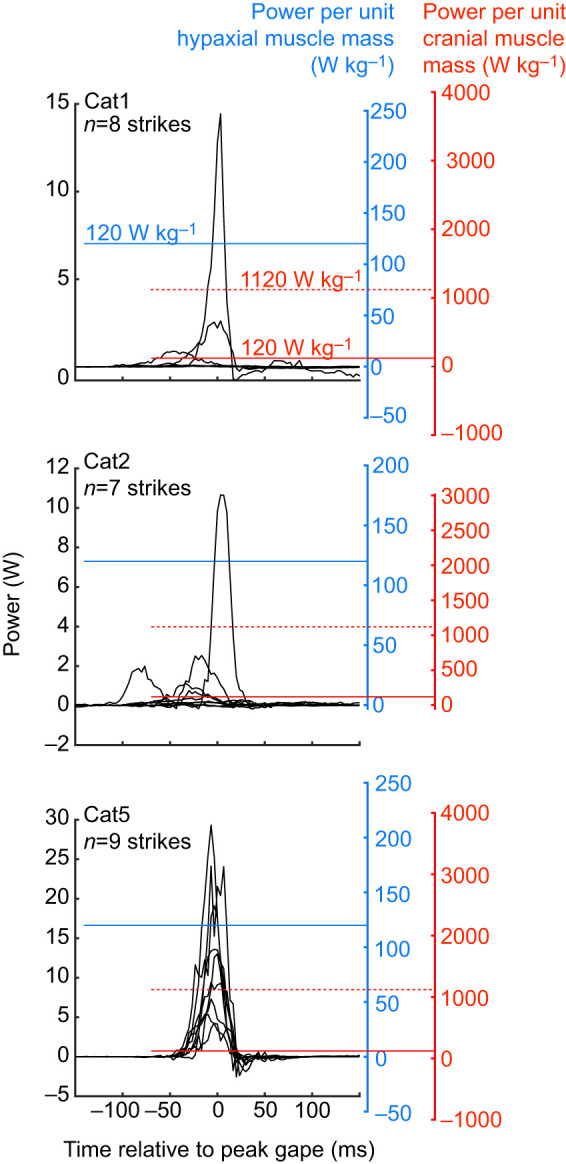
**Estimated mouth expansion power for each catfish.** The left-side (black) *y*-axis shows the absolute mouth expansion power for each strike from each catfish. The right-side *y*-axes show the power values for the same strikes, but relative to either the mass of the hypaxial muscles (blue) or the summed mass of all the cranial muscles (red). The maximum hypaxial muscle power output of 120 W kg^−1^ from another catfish species (Van Wassenbergh et al., 2007a) is shown on both of these right-side *y*-axes (red and blue solid lines). The maximum power output ever recorded from vertebrate muscles of about 1120 W kg^−1^ (Askew and Marsh, 2001) is shown on the red *y*-axis (red dashed lines).

Catfish strikes had maximum instantaneous expansion powers of 0.07–29.3 W (Fig. 6), as estimated from measurements of intra-oral pressure and rate of mouth volume change. The maximum sub-ambient intra-oral pressure achieved in any strike was similar for each individual (−31.7 kPa in Cat1, −26.7 kPa in Cat2 and −31.7 kPa in Cat5), although Cat5 had the greatest mean maximum sub-ambient pressure across all strikes (Table 1). We recorded the greatest absolute mouth volumes in Cat5 (Table 1), which achieved an estimated maximum mouth volume of 117 cm^3^, while Cat1 and Cat2 only reached maxima of 82.3 and 73.6 cm^3^, respectively. Similarly, the greatest rate of change in mouth volume during any strike was also observed in Cat5 (1234.8 cm^3^ s^−1^), while the maximum rates observed in Cat1 and Cat2 were only 502.3 and 655.2 cm^3^ s^−1^, respectively. When calculated relative to the mass of the hypaxial muscles, the estimated expansion power would have required hypaxial power outputs of no more than 246 W kg^−1^ (Fig. 6). This represents the peak power output required from the hypaxial muscles if they were the sole source of mouth expansion power, and only includes the bilateral hypaxial mass of the region over which shortening was measured. Epaxial muscle mass was not included as these muscles did not shorten during peak mouth expansion power. If estimated expansion power was calculated relative to the summed, bilateral mass of the cranial muscles, then mouth expansion would have required excessively high power outputs of up to 3663 W kg^−1^ (Fig. 6). For Cat5, all strikes would have required peak power outputs of over 500 W kg^−1^ from these head muscles if they were the sole source of mouth expansion power. But some of the low-power strikes recorded from Cat1 and Cat2 would have required only 11–100 W kg^−1^ peak power output from the cranial muscles (Fig. 6).

## DISCUSSION

**Fig. 7.**
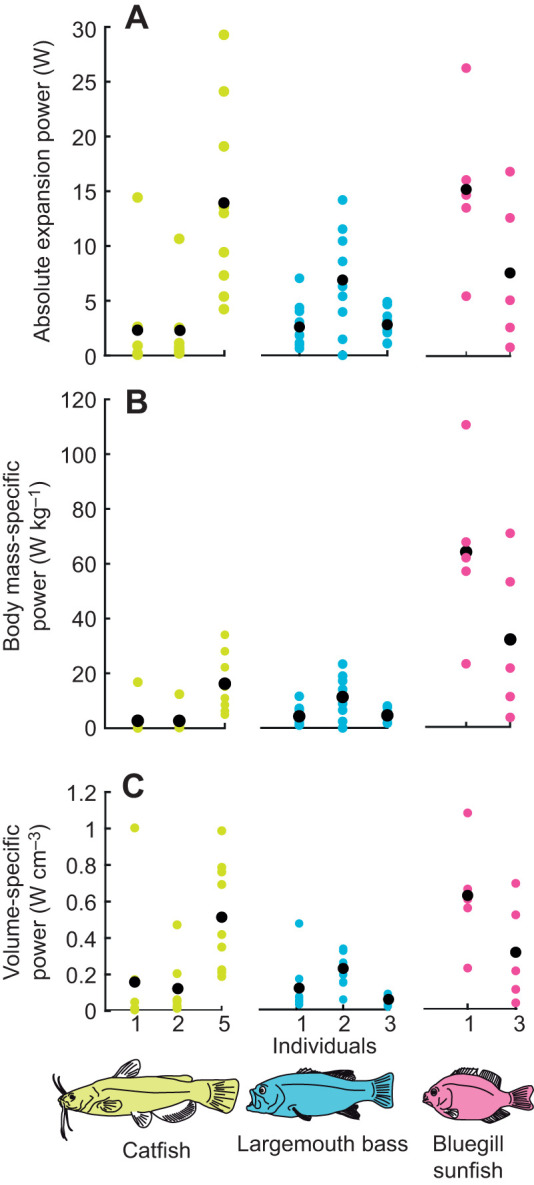
**Comparison of mouth expansion power estimated in channel catfish, largemouth bass (*Micropterus salmoides*) and bluegill sunfish (*Lepomis macrochirus*).** Data are shown from 2 or 3 individuals of catfish (yellow, *N*=24 strikes) from this study, bass (cyan, *N*=29 strikes) from Camp et al. (2015) and sunfish (magenta, *N*=11 strikes) from Camp et al. (2018). Colored circles represent data from individual strikes and black circles represent the mean of all strikes for a given individual. For all species, maximum estimated expansion power is shown as (A) the absolute magnitude of maximum power, (B) maximum power relative to the total body mass of each individual, and (C) maximum power relative to the maximum change in mouth volume for each strike.

We conclude that, under the feeding conditions recorded here, channel catfish do not use the epaxial muscles and cranio-vertebral interface as a motor during suction feeding. The epaxial muscles did not shorten during mouth expansion, and therefore did not generate power, and we observed little cranial elevation. Instead, the epaxial muscles and cranio-vertebral interface stabilized the head dorsally while the hypaxial muscles powered mouth expansion ventrally. Even without power from the epaxial muscles, the strikes from these channel catfish were equally powerful or more powerful than those of largemouth bass (Fig. 7), which do use both epaxial and hypaxial muscles to power mouth expansion (Camp et al., 2015). These results demonstrate how the axial muscles and postcranial interfaces can have different roles during suction feeding, with both motor and anchor functions contributing to powerful suction feeding.

### Cranio-vertebral interface as an anchor

Channel catfish did not use the cranio-vertebral interface as a motor, but as an anchor to stabilize the dorsal part of the cranial linkage and control the position of the head. The epaxial muscles did not shorten substantially during peak power: epaxial length changes were highly variable and often included lengthening, but on average the epaxial muscles maintained a relatively constant length (Fig. 4A). Without shortening, the epaxial muscles cannot generate positive velocity and power or elevate the neurocranium. Stabilizing the dorsal expansion system may be important for resisting negative pressure generated by ventral – and potentially lateral – expansion of the mouth cavity (Camp, 2019). Otherwise, the sub-ambient buccal pressures could rotate the cranium ventrally, resulting in ventral rotation of the entire head rather than expansion of the mouth cavity (Alexander, 1969; Carroll et al., 2004; Muller, 1987). As part of this anchor function, motion at the cranio-vertebral interface before the strike (Fig. 3) may also adjust the position of the head and mouth relative to the food, as previously hypothesized in clariid catfishes (Van Wassenbergh et al., 2006b). Anchoring of the cranio-vertebral interface is not surprising given observations of minimal cranial elevation in other catfish (Van Wassenbergh et al., 2009), but our study confirms this with direct measurements of *in vivo* muscle strain and bone motion.

We hypothesize that the epaxial muscles were actively, rather than passively, anchoring the cranio-vertebral interface, although this needs to be tested with electromyography. Theoretically, the cranio-vertebral interface could be anchored passively if the bones and joints of the interface (such as the Weberian apparatus; Fig. 1A) physically prevent dorsoventral motion. However, this seems unlikely for two reasons. First, although the timing and intensity may vary, the epaxial muscles are active during suction feeding across ray-finned fishes, including other ostariophysians with limited cranial elevation (Ballintijn et al., 1972; Grubich, 2001; Lauder, 1981; Sibbing, 1982; Wainwright et al., 1989). It would be surprising, therefore, if the epaxial muscles were completely inactive during suction feeding in channel catfish.

Second, the cranio-vertebral interface of channel catfish was capable of dorsoventral motion, despite the presence of the Weberian apparatus. In one individual (Cat5), we consistently observed small magnitudes of cranial elevation and depression (Fig. 3). This motion did not contribute substantially to mouth expansion – as only the initial few degrees of elevation occurred during the period of peak expansion power – but clearly such motions are possible. The same individual also showed small (2–4% *L*_i_) but consistent shortening in a subregion of epaxial muscle caudal to the Weberian apparatus (Fig. 4A). Varying magnitudes of cranial elevation have been observed in other ostariophysians, suggesting that the Weberian apparatus does not necessarily prevent dorsoventral motion between the head and body (Lauder, 1981; Van Wassenbergh et al., 2005a). Therefore, we hypothesize that in channel catfish the epaxial muscles were actively generating force (through isometric or eccentric contractions) to prevent dorsoventral motion and anchor the cranio-vertebral interface during suction feeding.

### Hyo-pectoral interface as a motor

The hypaxial muscles appeared to be the main source of mouth expansion power for channel catfish during suction feeding. Hypaxial muscle shortening extended beyond the cranial-most subregion (reported in Olsen et al., 2019), continuing to at least the level of the anal fin, and potentially further as we did not put markers in more caudal regions (Fig. 4B). In addition to hypaxial shortening, we found consistent caudoventral rotation (retraction) of the pectoral girdle relative to the body during peak expansion power (Fig. 3). Activity patterns of the hypaxial muscles have not been measured in this species during suction feeding, but these muscles are active during expansion in other suction feeding fishes (Lauder and Lanyon, 1980; Westneat, 2006). As we are unaware of plausible mechanisms for passive shortening of these muscles, it is reasonable to assume the hypaxial musculature was actively shortening to generate power.

Based on their mass, the hypaxial muscles in the region we measured could have powered all recorded strikes without exceeding power outputs of 250 W kg^−1^ (Fig. 6). Maximum hypaxial muscle power output has not been measured in channel catfish, but this is in line with the maximum power output measured from axial muscles in other fishes, e.g. 216 W kg^−1^ in largemouth bass (Coughlin and Carroll, 2006), 120 W kg^−1^ from clariid catfishes (Van Wassenbergh et al., 2007a), and below the 500 W kg^−1^ measured in saithe, *Pollachius virens* (Altringham et al., 1993). Of the muscles of the head, only the levator operculi and sternohyoideus shortened during peak expansion power and therefore could have contributed power (Fig. 5). However, these muscles are much smaller than the hypaxial muscles: the levator operculi was <0.5% and the sternohyoideus <4% of the mass of the hypaxial muscles. Even assuming they operated under optimal conditions for power production to reach power outputs of 300 W kg^−1^, together they could have generated no more than 3 W of power – only enough for the weakest strikes or about a tenth of the most powerful strike.

The shortening of the sternohyoideus in these channel catfish differed from some clariid catfishes, where the sternohyoideus lengthens or remains relatively isometric during mouth expansion (Van Wassenbergh et al., 2007b). Van Wassenbergh et al. (2007b) hypothesized that only relatively large sternohyoid muscles would be able to shorten against the hypaxials, otherwise shortening of the larger hypaxial muscles would be too forceful and the sternohyoid would be lengthened or remain isometric. However, in channel catfish (this study) and bluegill sunfish (Camp et al., 2018), the sternohyoid and hypaxial muscles both shorten even though the sternohyoideus muscle is <0.6% of body mass in the catfish and <1.5% of body mass in the sunfish. This suggests sternohyoid behavior may not depend only on its mass and the forces and motion applied to its attachment site on the cleithrum (by hypaxial shortening). In the species examined so far, the role of the sternohyoideus has been variable and investigating the role and mechanisms of sternohyoid length changes is an important subject for future studies.

### Relative power of catfish feeding strikes

Channel catfish do not seem to be limited to low-power strikes relative to fishes that use both epaxial and hypaxial muscles to power suction feeding. We compared channel catfish strikes with those of largemouth bass and bluegill sunfish, which are the only species for which similar muscle shortening and estimated mouth expansion power data are available. This is a very limited sample size, and it is unlikely that the maximum strike performance has been captured for any of these species, given the small number and laboratory conditions of recorded strikes (Astley et al., 2013). Therefore, the following are preliminary observations, intended to inform further hypotheses and future studies with more species. Additionally, catfish strikes were recorded feeding on less-elusive prey (worms, >25 mm), which may not have elicited as powerful strikes as the live goldfish (30–40 mm) prey used in the bass and sunfish studies. This suggests our current study may be underestimating the potential power of catfish suction feeding strikes compared with those of bass and sunfish.

The absolute magnitudes of peak instantaneous mouth expansion power in catfish strikes are similar to or greater than those of bass and sunfish (Fig. 7A). However, this does not take into account the different body and mouth sizes of these species. When standardized to total body mass, channel catfish and bass strikes occupy a similar range of mass-specific power, but bluegill sunfish strikes can be 2–3 times more powerful (Fig. 7B). In other words, sunfish can generate equally powerful strikes to those of bass and catfish, despite their much smaller body mass. If power is standardized to maximum mouth cavity volume – the space over which this power is being applied – channel catfish and bluegill sunfish strikes occupy a similar range and reach greater volume-specific power than largemouth bass strikes (Fig. 7C). Despite the potential for prey-type differences to lead to an underestimation of catfish suction power, all three comparisons suggest channel catfish strikes were not particularly low power. Interestingly, largemouth bass occupy the lowest range of strike power among the three species (Fig. 7), even though this species uses large regions of both epaxial and hypaxial muscles as motors.

Channel catfish could theoretically generate more powerful strikes if they used the epaxial muscles as motors, so why are these large body muscles not generating power during suction feeding? Suction feeding is a power-limited behavior, at least for centrarchids like largemouth bass and bluegill sunfish feeding on fish in the water column (Carroll and Wainwright, 2009). We presume this is also the case for channel catfish feeding benthically on worms, although it has not yet been tested. One hypothesis is that cranial elevation may not be an effective mechanism of expanding the mouth cavity in channel catfish because of their cranial morphology. Fish with relatively dorsoventrally flattened heads are expected to rely more on ventral than lateral expansion to increase the volume of the mouth cavity, based on the geometry of the hyoid apparatus (Alexander, 1970; Van Wassenbergh et al., 2009). However, these studies measured ventral expansion relative to the neurocranium, so presumably dorsoventral expansion could also be achieved by neurocranial elevation. The dorsoventrally flattened neurocranium of catfish would also result in unfavorable leverage – a long rostrocaudal out-lever relative to a short dorsoventral in-lever – for transmitting epaxial force (Carroll et al., 2004), although the epaxial muscles are clearly still capable of dorsoventral rotation of the neurocranium (Fig. 3).

Alternatively, the cranio-vertebral interface may be used to precisely position the mouth relative to the food to improve suction feeding performance, and cranial elevation would interfere with this function. This has previously been proposed for clariid catfishes (Van Wassenbergh et al., 2006b) to explain the variation in cranial elevation observed in these fishes during suction feeding. For benthic suction feeders like channel catfish, holding the mouth near the substrate may improve suction hydrodynamics (Nauwelaerts et al., 2007). Rotation of the neurocranium relative to the body may allow catfish to position the mouth relative to the food before the strike, while allowing space for ventral expansion of the mouth cavity (Van Wassenbergh et al., 2009). In support of this, some benthic, suction feeding sharks use minimal cranial elevation (Motta et al., 2002) and even cranial depression as in Fig. 3 (Camp et al., 2017). These hypotheses are not exclusive: geometrical and hydrodynamic factors could contribute to the cranio-vertebral interface acting as an anchor in channel catfish.

We expect that channel catfish are not unique, but that fishes with similar morphology and kinematics also use the cranio-vertebral interface as an anchor to position and stabilize the neurocranium during suction feeding. The absence of cranial elevation is likely to be a good proxy for the dorsal interface anchoring the head during feeding, with the force generated by passive musculoskeletal structures, active epaxial muscle contraction, or both. Suction feeding without cranial elevation seems common in catfishes (Van Wassenbergh et al., 2009), and other ostariophysians (but see Lesiuk and Lindsey, 1978). In such species, the hyo-pectoral interface is likely to be acting as a motor to power mouth expansion. Interestingly, a robotic model has demonstrated the kinematics and sub-ambient pressures of largemouth bass suction strikes can be replicated with the epaxial muscles functioning as an anchor (Kenaley and Lauder, 2016). This model shows the mechanism of mouth expansion does not require cranial elevation, although it did not include the power required for generating mouth expansion and sub-ambient pressure. We hypothesize that for all fishes, powerful suction feeding requires at least one of the axial muscles to act as a motor. Even for species that use elastic energy storage to amplify muscle power during suction feeding, the axial muscles seem to be an important source of energy to load the elastic system (Aerts et al., 1987; Van Wassenbergh et al., 2008). Therefore, the absence of cranial elevation in suction feeding fishes may also be a good indicator that the hypaxial muscles are functioning as motors. Conversely, the absence of pectoral girdle retraction and hypaxial muscle shortening would imply that the cranio-vertebral interface alone is powering suction feeding, although this has not yet been measured in any suction feeding fish to our knowledge.

### Conclusions

Channel catfish strikes in this study demonstrated how the epaxial muscles and cranio-vertebral interfaces of fishes can be used as a forceful anchor – and not just a powerful motor – during suction feeding. Our results suggest the axial muscles have important, but potentially variable roles in generating and controlling cranial motion during feeding. Future work can test this to determine whether the epaxial muscles actively anchor the cranio-vertebral interface, and function as brakes or struts rather than motors. While this range of muscle functions has been demonstrated during locomotion (Dickinson et al., 2000), it is less well established for feeding (but see Kenaley et al., 2019 and Konow et al., 2010). Thus, the axial muscles of suction feeding fishes may be a useful model for investigating the diversity of muscle functions in feeding systems. As the feeding functions of axial muscles are examined in more species, it will be possible to investigate the distribution of motor and anchor functions across fishes and how these roles may (or may not) correspond to morphology, body shape or feeding ecology. The morphological, phylogenetic and ecological diversity of ray-finned fishes offers an exceptional opportunity to study the mechanisms and evolution of the postcranial musculoskeletal systems' feeding roles.

## Supplementary Material

Supplementary information
