# Supplementary material for: Fishes can use axial muscles as anchors or motors for powerful suction feeding
Source: J Exp Biol. 2020 Sep 18;223(18):jeb225649. doi: 10.1242/jeb.225649 (PMC7520451; doi:10.1242/jeb.225649)
Supplement: Supplementary information [file jexbio-223-225649-s1.pdf]

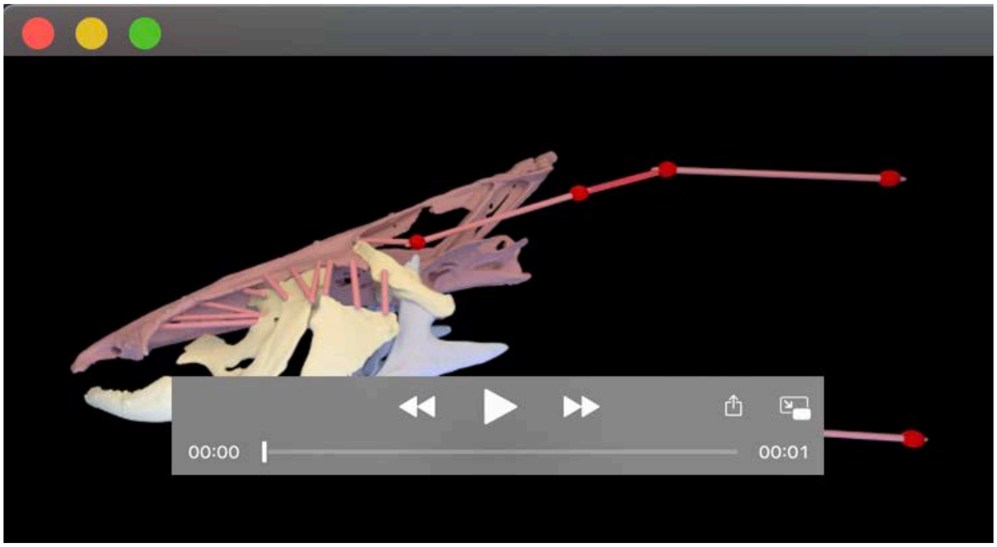


**Movie 1 – XROMM skeletal animation of a channel catfish (*Ictalurus punctatus*, individual Cat5) strike, with muscle length measurements visualized as pale red cylinders.** The neurocranium (red), cleithrum (blue), and left-side lower jaw, suspensorium, operculum, urohyal, and hyoid bar are shown in an antero-lateral view, moving relative to the body plane, i.e., the fish's body. For the levator arcus palatini, dilator operculi, levator operculi, and sternohyoideus, the three representative fibers are shown extending between bony origin and insertion sites. The lengths of these muscles were measured from the XROMM animation. Dorsal (epaxial) and ventral (hypaxial) muscle lengths were measured from intramuscular markers (red spheres) using fluoromicrometry. The lengths of each subregion of the epaxials and hypaxials are shown as pale red cylinders. The animation was created from X-ray videos filmed at 300 Hz, but it has been slowed down about 12 times in this movie.

Journal of Experimental Biology • Supplementary information

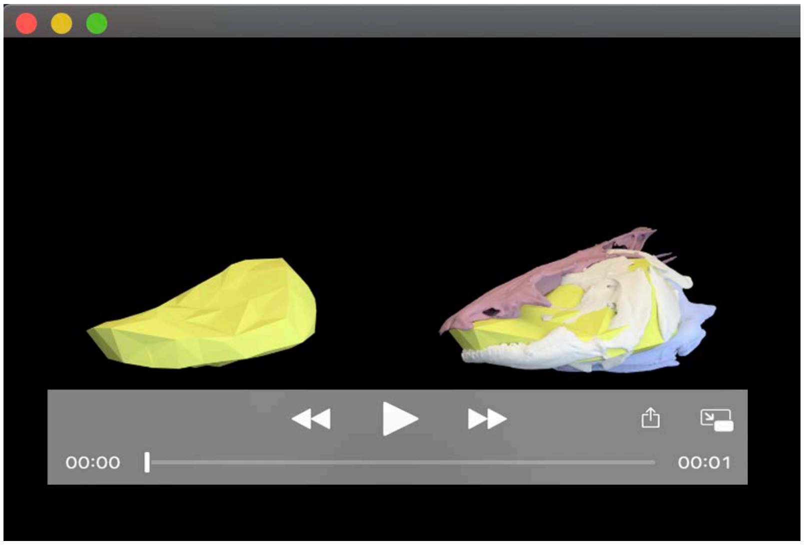


**Movie 2– Dynamic digital endocast of the mouth cavity of a channel catfish (*Ictalurus punctatus*, individual Cat5) during a suction feeding strike.** The endocast, shown by itself (left) and with the skeletal animation (right) was used to calculate the volume of the mouth cavity at each frame (see Methods for details), moving relative to the body plane, i.e., the fish's body. The animation was created from X-ray videos filmed at 300 Hz, but it has been slowed down about 12 times in this movie.

Journal of Experimental Biology • Supplementary information
